# Mutational Profile and Clonal Evolution of Relapsed/Refractory Diffuse Large B-Cell Lymphoma

**DOI:** 10.3389/fonc.2021.628807

**Published:** 2021-03-11

**Authors:** Boram Lee, Hyunwoo Lee, Junhun Cho, Sang Eun Yoon, Seok Jin Kim, Woong-Yang Park, Won Seog Kim, Young Hyeh Ko

**Affiliations:** ^1^Samsung Genome Institute, Samsung Medical Center, Sungkyunkwan University School of Medicine, Seoul, South Korea; ^2^Department of Health Science and Technology, Samsung Advanced Institute for Health Sciences and Technology, Sungkyunkwan University, Seoul, South Korea; ^3^Department of Pathology and Translational Genomics, Samsung Medical Center, Sungkyunkwan University School of Medicine, Seoul, South Korea; ^4^Division of Hematology and Oncology, Department of Medicine, Samsung Medical Center, Sungkyunkwan University School of Medicine, Seoul, South Korea; ^5^Department of Molecular Cell Biology, Sungkyunkwan University School of Medicine, Suwon, South Korea

**Keywords:** refractory diffuse large B-cell lymphoma, relapsed diffuse large B-cell lymphoma, chemotherapy resistance, tumor evolution, immune evasion, prognostic marker, next-generation sequencing (NGS)

## Abstract

Primary refractory/relapsed diffuse large B-cell lymphoma (rrDLBCL) is an unresolved issue for DLBCL treatment and new treatments to overcome resistance is required. To explore the genetic mechanisms underlying treatment resistance in rrDLBCL and to identify candidate genes, we performed targeted deep sequencing of 430 lymphoma-related genes from 58 patients diagnosed with rrDLBCL. Genetic alterations found between the initial biopsy and biopsy at recurrence or refractory disease were investigated. The genes most frequently altered (> 20%) were (in decreasing order of frequency) *CDKN2A, PIM1, CD79B, TP53, MYD88, MYC, BTG2, BTG1, CDKN2B, DTX1, CD58, ETV6*, and *IRF4*. Genes mutation of which in pretreatment sample were associated with poor overall survival included *NOTCH1*, *FGFR2*, *BCL7A*, *BCL10*, *SPEN* and *TP53* (*P* < 0.05). *FGFR2, BCL2*, *BCL6*, *BCL10*, and *TP53* were associated with poor progression-free survival (*P* < 0.05). Most mutations were truncal and were maintained in both the initial biopsy and post-treatment biopsy with high dynamics of subclones. Immune-evasion genes showed increased overall mutation frequency (*CD58, B2M*) and variant allele fraction (*CD58*), and decreased copy number (*B2M, CD70*) at the post-treatment biopsy. Using the established mutational profiles and integrative analysis of mutational evolution, we identified information about candidate genes that may be useful for the development of future treatment strategies.

## Introduction

Diffuse large B-cell lymphoma (DLBCL) is a heterogeneous disease comprising distinct types of aggressive B-cell lymphoma with different biology and clinical outcomes. Most of the patients with DLBCL respond well to standard immunochemotherapy, but 10% of patients present with primary refractory disease and an additional 30–40% experience relapse following an initial response to therapy (1, 2). Patients with primary refractory or relapsed disease require a new treatment modality to overcome the resistance to treatment, but this remains an unmet need in the management of patients with DLBCL (2, 3).

DLBCL is divided into two distinct molecular subtypes classified according to the gene expression profile (4). The germinal center B-cell-like (GCB) subtype is characterized by mutation of *EZH2*, translocation of *BCL2*, *BCL6*, or *MYC*, and activation of the phosphatidylinositol 3 kinase (PI3K)-Akt-mTOR signaling pathway; genes that function in the normal germinal center are expressed by this subtype (5, 6). The activated B-cell-like (ABC) subtype is characterized by the constitutive activation of the NF-κB signaling pathway and mutation in the genes engaging in the B-cell receptor (BCR) signaling and/or toll-like receptor signaling pathways. The ABC subtype involves mutations in *TNFAIP3*, *CARD11*, *MYD88*, *BCL10*, *MALT1*, and *BCL6*, and results in activation of the transcription factor NF-κB (7, 8).

The differences in these intracellular oncogenic signaling pathways have prognostic significance and can be exploited for therapeutic benefit. A recent study showed that these molecular subgroups based on the cell of origin of DLBCL can be further divided into five clusters based on mutations, somatic copy number alterations (SCNAs), and structural variants (SVs) of the genome (9). Each ABC-type and GCB-type DLBCLs could be classified into two subclasses showing different survival outcomes and a class featuring *TP53* mutation had a moderate survival outcome. DLBCLs belonging to the ABC-type DLBCL can be classified into two molecular groups. One molecular group is characterized by gain of 18q and overexpression of *BCL2*, and mutations of *CD79B* and *MYD88* L265P. The other ABC-type molecular group harbors translocation of *BCL6* and mutations of genes involved in the NOTCH and NF-κB signaling pathways. GCB-type DLBCL can also be divided into two molecular groups. One molecular group is characterized by translocation and mutation of *BCL2* with mutations of the epigenetic regulators *IRF8* and *TNFRSF14*, and the other molecular group has mutations in linker histone genes with mutations of *CD58*, *RHOA*, *CARD11*, *BRAF*, and *STAT*.

The number of therapeutic agents targeting genetic variations and related signaling pathways is increasing as the numbers of discovered mutations associated with initiation, transformation, and progression increase. Novel therapeutic agents that target the BCR pathways include dasatinib (Lyn inhibitor), ibrutinib (BTK inhibitor), fostamatinib (SYK inhibitor), or enzastaurin (PKCβ inhibitor) (10–13). Overexpression of myc, through translocation or by other mechanisms can be targeted indirectly by epigenetic manipulation with a BET bromodomain inhibitor. Histone deacetylase inhibitors (e.g. vorinostat) hold promise for lymphomas with *CREBBP* or *EP300* mutations (14).

Because recently developed novel therapies act on tumors through mechanisms involving genetic alterations, it seems worthwhile to study whether specific genetic alterations can predict a poor response to current immunochemotherapy. In this study, we performed targeted deep sequencing of 430 lymphoma-related genes for 58 patients diagnosed with relapsed/refractory DLBCL (rrDLBCL). Various mutations, SCNAs, and SVs were investigated in a sample obtained in the initial biopsy and that obtained at the time of relapse or diagnosis of refractory disease. We also analyzed samples from 15 patients with a good response to conventional immunochemotherapy as a control and compared the data between these controls and those with rrDLBCL.

## Materials and Methods

### Patient Selection

Patients with rrDLBCL diagnosed between 1 January 2002 and 31 December 2018 in the Samsung Medical Center were enrolled in the study. Primary refractory DLBCL was defined as progression of the disease during initial R-CHOP treatment without a complete remission (CR) or relapse of the disease after a transient CR in < 6 months from the end of the initial therapy. Relapsed DLBCL was defined as DLBCL that reappeared after CR lasting > 6 months. If the DLBCL relapsed > 60 months after the treatment, we called it a late relapse. Sixteen of the 74 rrDLBCL patients were diagnosed with transformed DLBCL or primary CNS DLBCL and were excluded. For comparison, 15 patients with DLBCL who had been cured with a follow-up period of > 6 years were included as controls. All patients were treated with initial R-CHOP immunochemotherapy with or without radiotherapy or stem cell transplantation. This study was approved by institutional review board of Samsung Medical Center (IRB 2013-12-076-005) in accordance with the tenets of the Declaration of Helsinki.

### Clinical Data

The revised International Prognostic Index (IPI) score (15) was calculated by counting the number of the following risk factors for each patient: 1) age > 60 years at the time of diagnosis, 2) stage III or IV disease, 3) elevated serum LDH level, 4) Eastern Cooperative Oncology Group performance status > 2, and 5) > 1 extranodal site. An IPI score of 0–2 is defined as low risk and 3–5 is defined as high risk.

### Immunohistochemistry

Formalin-fixed, paraffin-embedded specimens were used in the ancillary study. Immunohistochemical staining of 4-μm paraffin sections were performed using a Bond Max automated immunostainer (Leica Biosystems, Melbourne, Australia). Monoclonal antibodies against CD20 (L26, 1/200; Dako, Glostrup, Denmark), CD3 (polyclonal, 1/200; Dako), CD10 (56C6, 1/250; Novocastra, Newcastle upon Tyne, UK), BCL6 (LN22, 1/80; Novocastra), MUM1 (MUM1p, 1/500; Dako), BCL2 (124, 1/100; Dako), and Myc (Y69, cat:ab32072, 1/100; Abcam, Burlingame, CA, USA) were used. The cell of origin (COO) subtype was determined using the Hans algorithm (16).

### Fluorescence *In Situ* Hybridization Analysis of MYC, BCL2, and BCL6 Translocation

Interphase fluorescence *in situ* hybridization (FISH) analysis was performed for all samples using a Vysis LSI^®^ MYC Dual Color Break Apart Rearrangement Probe (Abbott/Vysis, Des Plains, IL, USA) for detection of *MYC* rearrangement, a Vysis LSI^®^ BCL2 Dual Color Break Apart Rearrangement Probe (Abbott/Vysis) for detection of *BCL2* rearrangement and a Vysis LSI^®^ BCL6 Dual Color Break Apart Rearrangement Probe (Abbott/Vysis) for detection of *BCL6* rearrangement. The determined cut-off value for the detection of a rearrangement of *MYC*, *BCL2*, and *BCL6* was 5%.

### Targeted Panel Sequencing

Targeted panel sequencing was performed using a HemaSCAN panel that contained 430 genes related to hematological malignancies. Genomic DNA was extracted using a QIAamp DNA Mini kit (Qiagen, Valencia, CA, USA), according to the manufacturer’s protocol. DNA quality and quantity were analyzed using a Nanodrop 8,000 UV–Vis spectrometer (NanoDrop Technologies, Wilmington, DE, USA), Qubit 2.0 Fluorometer (Life Technologies, Carlsbad, CA, USA), and 2200 TapeStation Instrument (Agilent Technologies, Santa Clara, CA, USA). Genomic DNA was sheared using a Covaris S220 instrument (Covaris, Woburn, MA, USA). Target capture was performed using the SureSelect XT Reagent Kit, HSQ (Agilent Technologies), and a paired-end sequencing library was constructed with a barcode. Sequencing was performed on a HiSeq 2500 with 100-bp reads (Illumina, San Diego, CA, USA). The paired-end reads were aligned to the human reference genome (hg19) using BWA-MEM v0.7.5. Samtools v0.1.18, GATK v3.1-1, and Picard v1.93 were used for BAM file handling, local realignment, and removal of duplicate reads, respectively.

Single nucleotide variants (SNVs) with a variant allele fraction (VAFs) > 1% were detected using MuTect v1.1.4 (17), and Lofreq v0.6.1 (18). Sequencing errors were filtered out by an in-house algorithm using data extracted from each BAM file (19). Small insertions and deletions (indels) < 30 bp in size were detected using Pindel v0.2.5a4 (20). Possible germline polymorphisms were also filtered out if the allele frequency was > 0.1% in any of the normal population databases including the Genome Aggregation Database (21), Korean Reference Genome Database, or Korean Variant Archive (22). SVs and large indels > 30 bp in size were detected using JuLI (23). SCNAs of each gene were also detected using an in-house copy number caller with copy numbers > 6 being marked as amplifications and copy numbers < 0.8 designated as deletions. The tumor mutational burden (TMB) was calculated by counting the number of SNVs and indels, and converting the value to count per megabase pairs.

### Statistical Analysis

Statistical analysis was conducted using R-3.6.1. Continuous variables were compared between two groups using Student’s *t* test, and categorical variables were compared using the chi-square test. Progression-free survival (PFS) was calculated from the date of diagnosis to the date of disease progression or relapse. Overall survival (OS) was calculated from the date of diagnosis to the date of death. Mutational frequency of each gene was compared using logistic regression analysis corrected for COO. Any type of alteration, including SNVs, indels, SCNAs, and SVs, were counted to calculate the mutational frequency. To calculate the hazard ratio (HR) and *P* value, the Cox proportional-hazards model corrected for COO and IPI score was used. *P* values were adjusted using the Benjamini and Hochberg method. A false discovery rate (FDR) < 0.1 was considered to be significant.

### Pre- and Post-Chemotherapy Paired-Sample Analysis

The VAF values of the SNV/indel and copy number of amplified or deleted genes were used to compare clonal changes in the pre- and post-chemotherapy samples for each patient. The VAF value is not directly comparable because it differs according to the purity. To adjust for differences in VAF between patients, the VAF values of mutations shared between pre- and post-chemotherapy samples were fitted using robust linear regression. Mutations with VAF values between 0.4 and 0.6 in both the pre- and post-therapy samples and > 0.1 apart from the fitted value were considered as germline mutations and excluded. To summarize the differences in VAF values between patients, fitted VAF values were normalized using the following equation to maintain the median VAF value as 0.5:

$$Normalized\;VAF=\frac{V\times (1-m)}{V\times (1-m)+(1-V)\times m}$$

where V is the fitted VAF value and m is the median of the fitted VAF value.

Only samples with calculated tumor purity >60% were used in the analysis to accurately compare the copy number of genes. For all genes with copy number ≥ 3 or ≤ 1, the changes in copy number between pretreatment and post-treatment samples was analyzed.

### Molecular Classification

The data of Chapuy et al. (9) were retrained with a lasso regression model using 77 of 105 features that were available from our panel sequencing and FISH results. Among the 77 features, 71 features had non-zero coefficient. Cluster 0 was not used because it represented extremely low-purity samples and more than one alteration was used for modelling in all of our samples. When tested using the leave-one-out cross-validation, the overall accuracy of the adjusted model was 0.75 compared with the original classification (**Supplementary Table 1**). When applying this model, copy number changes are aggregated for each chromosomal band using the mean copy number of the included genes. For a mean copy number > 2.2 and > 3.7, amplification scores of 1 and 2 were given, respectively. For a mean copy number < 1.6 and < 1.1, deletion scores of 1 and 2 were given, respectively.

We slightly modified the seed classification of Schmitz et al. (8) and used the presence of the final features of the seed genes to assign class. Those with *MYD88* L265P mutation or *CD79B* SNV/indel or amplification were classified as MCD, those with *BCL6* fusion or *NOTCH2* mutation or amplification were classified as BN2, those with *NOTCH1* mutation were classified as N1, and those with *BCL2* fusion or *EZH2* mutation were classified as EZB.

## Results

### Patient Characteristics

Details of the patients and their clinicopathological characteristics are summarized in **Supplementary Table 2**.

**Figure 1:**
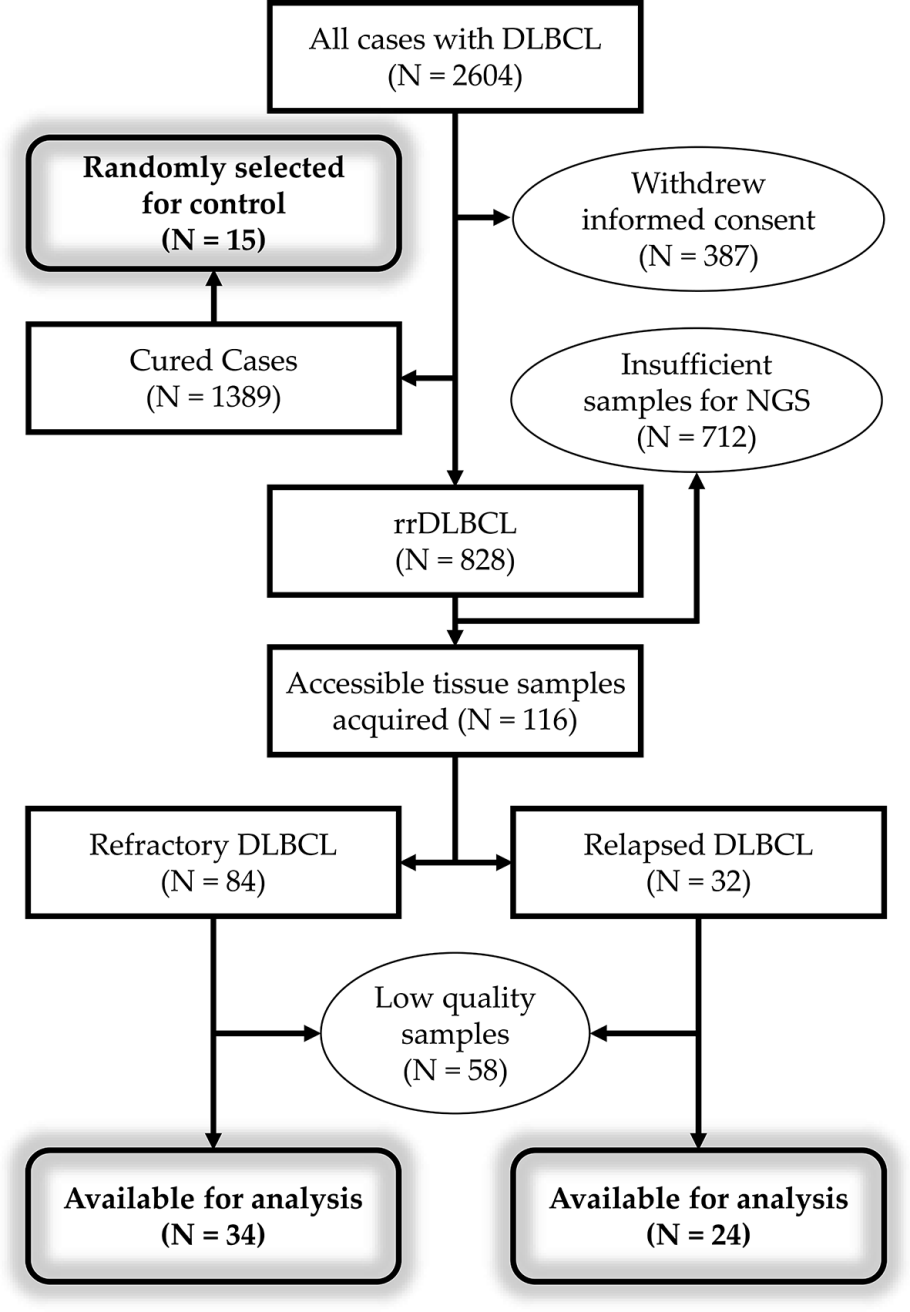
Workflow of patient selection. Total of 2,604 patients were diagnosed with DLBCL. Of the 2,217 patients with informed consent to review of medical records, 828 patients have refractory or relapsed disease. DNA extraction was performed using samples from 116 patients with rrDLBCL, but samples from 58 patients failed to pass the quality control. DLBCL, diffuse large B-cell lymphoma; rr, refractory/relapsed; NGS, next-generation sequencing.

A total of 58 patients were included (**Figure 1**): 34 patients with refractory DLBCL and 24 patients with relapsed DLBCL. For comparison, we also included the 15 cured patients who exhibited CR and no subsequent relapse. The median follow-up durations were 16.6 months (range, 5.4–62.7) for refractory DLBCL, 62 months (range, 20.4–200.5) for patients with relapsed DLBCL, and 87.5 months (range, 74.9–102) for cured patients. Among the 24 patients with relapsed DLBCL, the median time to relapse was 17.8 months (range, 10.8–56.1) in 17 patients; the other six patients had a very late relapse with a median time to relapse of 107.05 months (range, 97–182).

**Figure 2:**
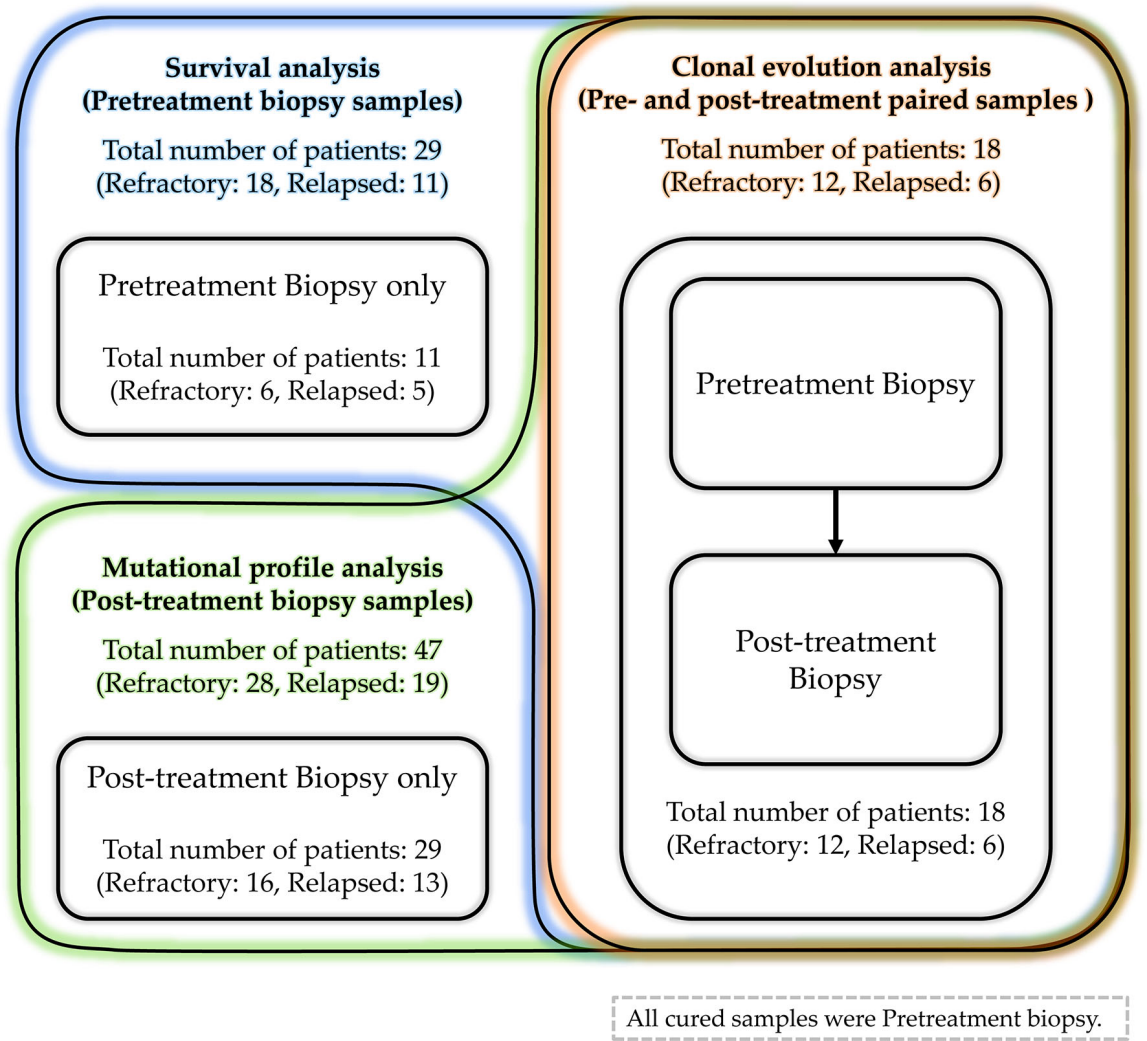
The details of acquired samples. The pretreatment biopsy samples were obtained from 29 patients (18 refractory DLBCL and 11 relapsed DLBCL). Among them, samples obtained from 18 patients (12 refractory DLBCL and 6 relapsed DLBCL) were paired samples and other samples from 11 patients (6 refractory DLBCL and 5 relapsed DLBCL) were unpaired pretreatment samples. The post-treatment biopsy samples were obtained from 47 patients (28 refractory DLBCL and 19 relapsed DLBCL). Excluding paired samples from 18 patients, samples from 29 patients (16 refractory DLBCL and 13 relapsed DLBCL) were unpaired post-treatment samples.

The COO did not differ significantly between patients with refractory, early relapse, and cured DLBCL. The IPI score was high, indicating poor prognosis in patients with refractory disease or early relapse compared with those with late relapse or cured DLBCL (*P* = 0.006). *MYC* translocation was found in 19% of early relapsed and 30% of refractory DLBCL patients. None of the late relapsed case had *MYC* translocation. Only one patient with refractory disease was found to have rearrangement of *MYC* and *BCL6*. Of the 58 patients with rrDLBCL, diagnostic pretreatment biopsies and post-treatment biopsies obtained after the progression of tumor were available for 29 patients and 47 patients, respectively. Pairs of pre- and post-treatment biopsy data were available for 18 patients (**Figure 2**).

### Mutational Profile of rrDLBCL

**Figure 3:**
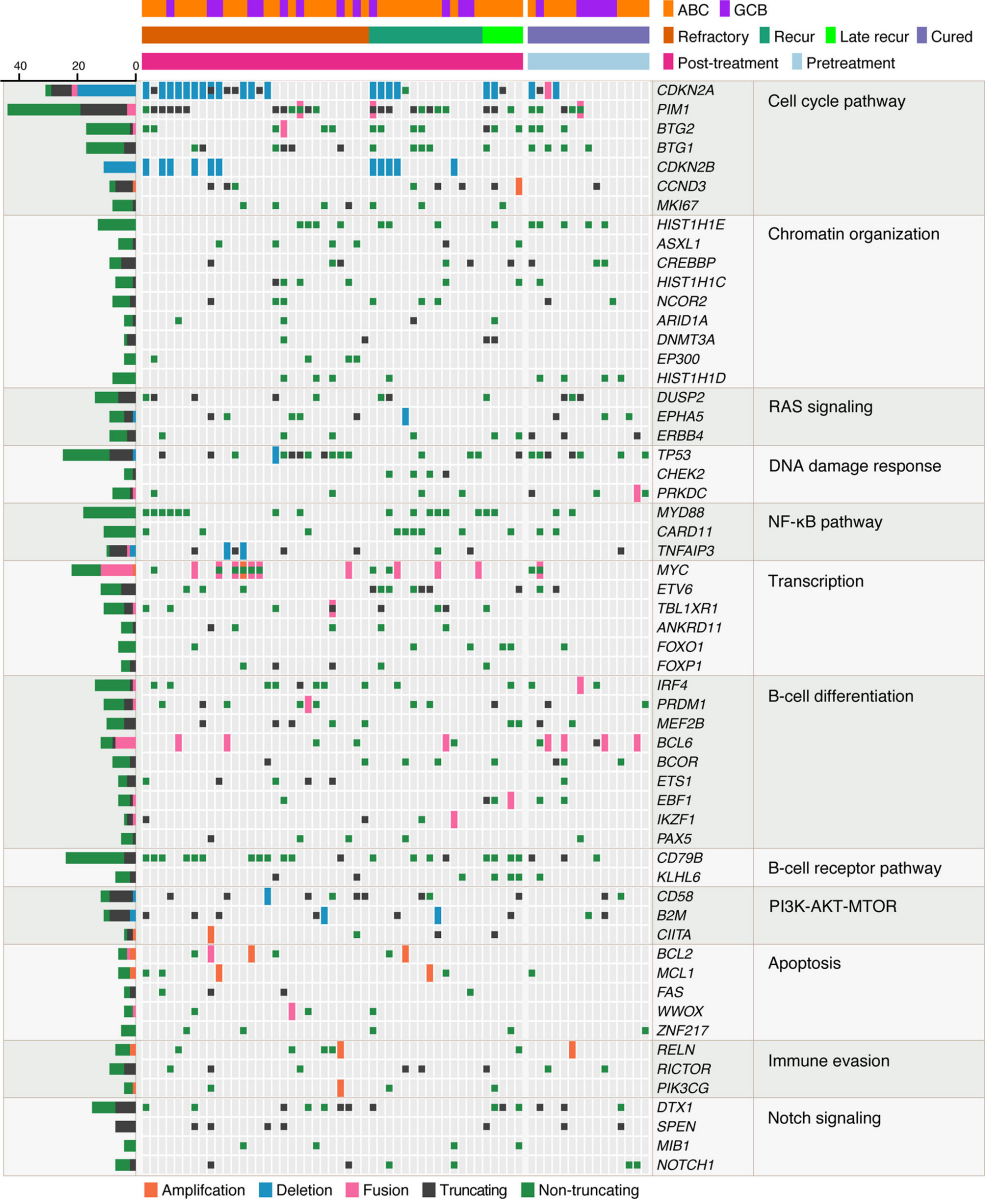
Mutational profiles of post-treatment tumor samples of relapsed/refractory diffuse large B-cell lymphoma (rrDLBCL) and pretreatment tumor samples of cured DLBCL. rrDLBCL patients include 28 refractory, 14 early recurred, and 5 late recurred patients. Genes mutated in > 3 samples and pathways having > 1 mutated genes are shown. The pathways and genes are ordered by the frequency of mutation. Copy numbers > 6 are marked as amplifications and copy numbers < 0.8 are marked as deletions. COO, cell of origin; ABC, activated B-cell-like subtype; GCB, germinal center B-cell-like subtype.

The mutational profiles of post-treatment biopsy results for 47 rrDLBCL patients are illustrated in **Figure 3**. At least five non-synonymous single nucleotide variants SNVs, SVs, or SCNAs were detected in rrDLBCL tumor samples (median, 17; range, 5–44).

**Figure 4:**
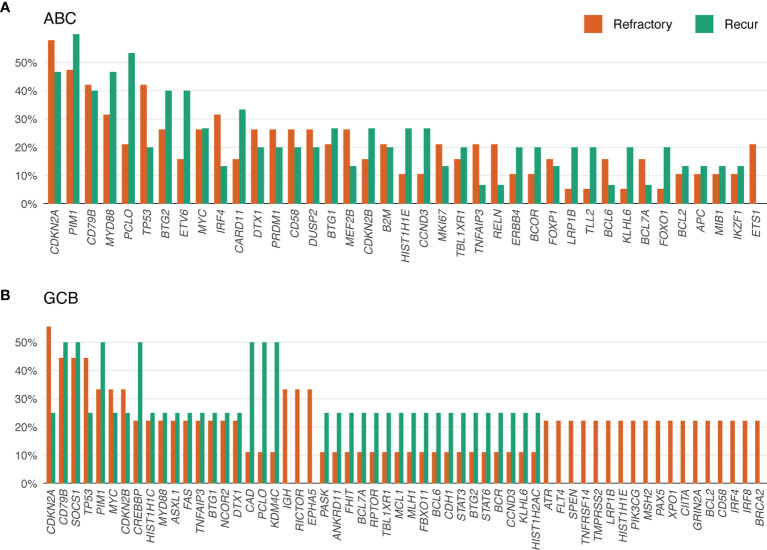
Mutational frequency of genes in post-treatment tumor samples from patients with relapsed/refractory diffuse large B-cell lymphoma (rrDLBCL). **(A)** The mutational frequency ABC-type rrDLBCL including 19 refractory and 15 recurred patients. **(B)** The mutational frequency of GCB-type rrDLBCL including 9 refractory and 4 recurred patients.

**Figure 5:**
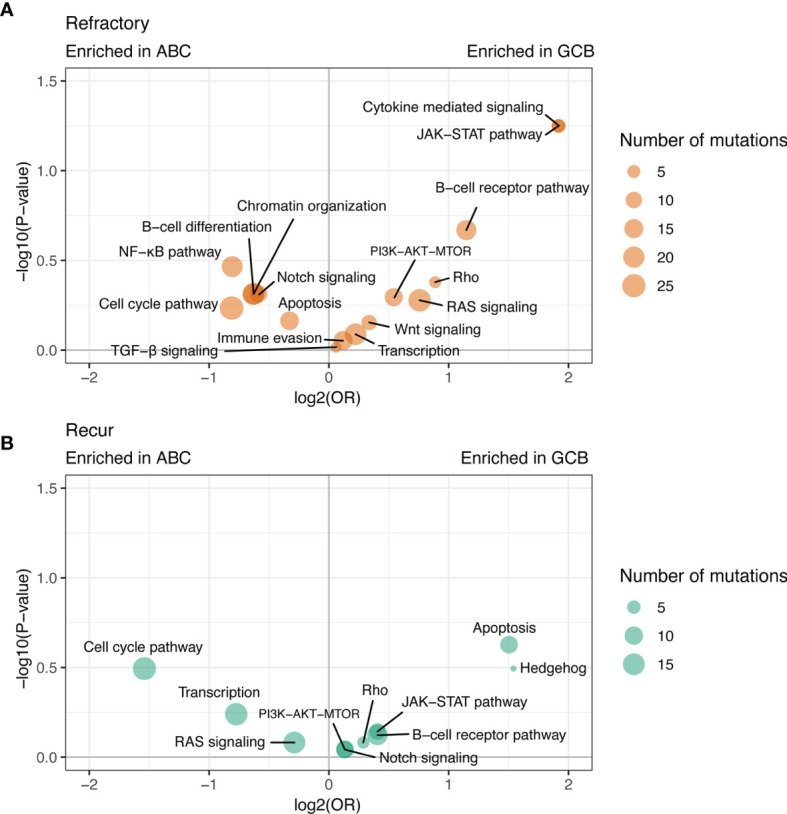
Differences in the frequency of mutated pathways between post-treatment tumor samples of the ABC-type rrDLBCL and GCB-type rrDLBCL. Mutations of genes are aggregated for each pathway and the differences between ABC-type rrDLBCL and GCB-type rrDLBCL are shown for **(A)** refractory disease and **(B)** recurred disease.

The genes most frequently altered (> 20% of patients), in decreasing order of frequency, were *CDKN2A*, *PIM1*, *CD79B*, *TP53*, *MYD88*, *PCLO*, *MYC*, *BTG2*, *BTG1*, *CDKN2B*, *DTX1*, *CD58*, *ETV6*, and *IRF4*. Mutations were especially frequent in *MYD88* [Present study (pretreatment), 35%; Present study (post-treatment), 34% vs. Karube et al. (pretreatment), 23%; Morin et al. (post-treatment), 20%; COSMIC (pre- or post-treatment), 15%; cured, 13%], *CD79B* (41% and 43% vs. 9, 16, 4, and 20%, respectively), *CDKN2A* (38% and 51% vs. 26, not applicable, 19, and 27%, respectively), and *MYC* (35% and 27% vs. 9, 12, 4, and 13%, respectively) than for that reported for DLBCL previously (24–26) and in the cured group in our study (**Supplementary Table 3**). Mutations in *PRDM1*, *MKI67*, *MYD88*, and *IRF4* tended to occur more frequently in patients with ABC-type DLBCL. By contrast, mutations in *SOCKS1*, *CREBBP*, *NCOR2*, *RICTOR*, *PAX5*, and *BCL2* were more frequent in patients with GCB-type DLBCL (**Figures 4** and **5**, **Supplementary Table 4**).

We examined the molecular functions and relevant signaling pathways of mutated genes. In patients with rrDLBCL, mutation of more than one gene that plays a role in the cell cycle pathway was found in 92%, in the chromatin remodeling in 81%, in the RAS signaling pathway in 81%, in the NF-κB pathway in 79%, in the DNA damage response in 79%, in transcription regulation in 79%, in the B-cell differentiation in 75%, in the BCR pathway in 64%, and in the immune evasion in 51% (**Supplementary Table 4**). The NOTCH and JAK–STAT pathways were affected in 45% and 30% of these patients, respectively. *DTX1* was the most frequent mutation (23%) among genes involved in the NOTCH pathway followed by *SPEN* (11%), *NOTCH1* (9%) *NOTCH2* (4%), and *SGK1* (2%). *DTX1* is a negative regulator of NOTCH signaling (27) and a predictor of worse prognosis. *SOCS1*, a negative regulator of the receptor-signaling pathway *via* JAK–STAT was mutated in 15% of the rrDLBCL patients.

Tumor-suppressor genes involved in cellular proliferation and the DNA damage response were mutated at a high frequency and included *CDKN2A* (51%), *CDKN2B* (23%), *BTG2* (28%), *BTG1* (23%), and *TP53* (34%). The tumor-suppressor genes *CDKN2A* and *TP53* are two of the most frequently inactivated genomic loci in human cancers (28, 29). As expected because of its tumor-suppressor function, *CDKN2A* was altered by inactivating mutations including homozygous deletion (18/27 alterations), truncating mutation (6/27), non-truncating mutation (2/27), and SV (1/27). rrDLBCL harboring either the *CDKN2A* (24 patients) or *TP53* (16 patients) alteration occurred in 37 (79%) patients, and each mutation was exclusive to the other except for three patients.

*BTG2* and *BTG1* are tumor-suppressor genes and members of the human BTG/TOB family. BTG2 is a p53-dependent component of the DNA damage cellular response pathway (30) and has been shown to negatively control a cell cycle check-point at the G1 to S phase transition (31). *BTG2* was altered by a truncating mutation (1/14 alterations), non-truncating mutation (12/14), and SV (1/14). BTG1 was affected by a truncating mutation (4/13 alterations) and non-truncating mutation (9/13) and overlapped with a mutation of either *CDKN2A* or *TP53* (**Supplementary Table 5**).

Mutations of oncogenes involved in the regulation of cell cycle and transcription, and activation of the BCR-NF-κB pathway included *PIM1* (49% of patients), *CD79B* (43%), *MYD88* (34%), *MYC* (28%), *CARD11* (19%), and *ETV6* (21%). *MYC* alterations comprised focal amplification (1/19 alterations), non-truncating mutation (8/19), and SV (10/19). All variants of *MYD88* mutation were *MYD88* L265P. *PIM1* mutation comprised many different variants in each sample (median, 0; range, 0–15) and 48% were SNV/indel. Aberrant somatic hypermutation is an important molecular feature of DLBCL and targets several proto-oncogenes (32, 33). When analyzed using a method published previously (32), among the genes mutated in these rrDLBCL patients, *PIM1* and *BTG1* had an SHM indicator value of < 0.1, which confirmed the alterations by somatic hypermutation (**Supplementary Table 6**).

There were no significant differences in the rates of mutation between patients with refractory DLBCL and relapsed DLBCL. The overall mutation frequency did not differ significantly between the initial biopsy and post-treatment biopsy. The frequencies of mutations involving immune evasion (*B2M*, *CD58*), the NF-κB pathway (*CARD11*, *TBL1XE1*), the JAK–STAT pathway (*SOCS1*), chromatin remodeling (*CREBBP*, *DNMT3A*), and the cell cycle (*CDKN2A*, *PIM1*) were higher in the post-treatment biopsy, although the difference was not significant (**Supplementary Table 3**).

### Prognostic Significance of Genes and Pathways Mutated in the Pretreatment Biopsy

The mutational frequency of genes and pathways in pretreatment biopsy for refractory, recurred and cured DLBCL were shown in **Supplementary Table 7**. Alterations in B-cell differentiation pathway and JAK–STAT pathway tended to be more frequent in cured DLBCL (86.7% and 66.7%) than recurred DLBCL (45.5% and 18.2%).

Pretreatment biopsy samples were available for 29 patients with rrDLBCL, including 18 with refractory DLBCL, eight with early relapse, and three with late relapse. Twenty-six patients died and three patients were alive with the disease at the follow-up. The median OS was 20.6 months (range, 5.4–200.5) and median PFS was 9.1 months (range, 2.3–182.0).

**Figure 6:**
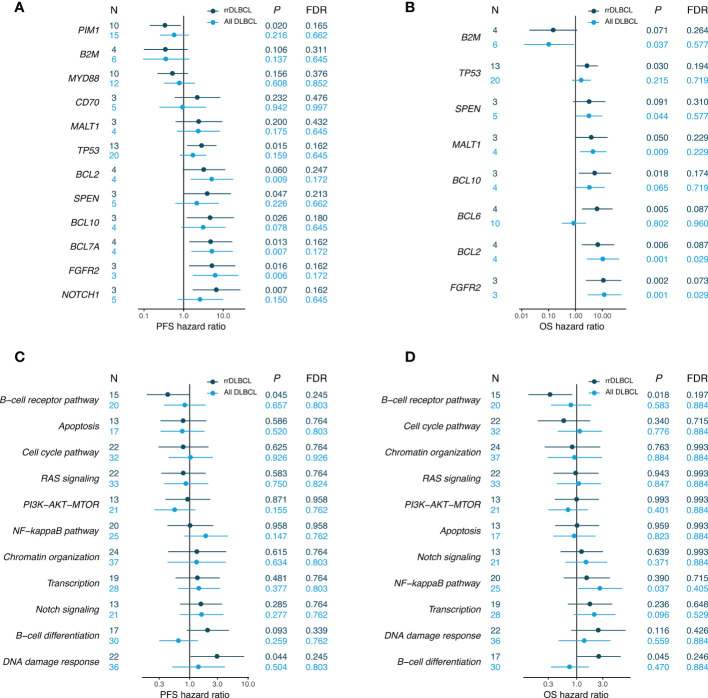
Associations between survival probability and mutations in pretreatment tumor samples. Hazards ratios (HRs) are shown separately for pretreatment samples of rrDLBCL and all DLBCL including cured disease. HR and *P* values were corrected by the COO and IPI score. **(A, B)** Genes with *P* values < 0.05 in the univariate analysis for rrDLCL are included. **(C, D)** Pathways with > 9 mutated rrDLBCL samples are included.

We used multivariate analysis to evaluate the prognostic significance of genes mutated in > 3 patients by considering the IPI and COO. Mutated genes associated with a poor OS included *FGFR2*, *BCL2*, *BCL6*, *BCL10*, and *TP53* (*P* < 0.05). Mutations in *NOTCH1*, *FGFR2*, *BCL7A*, *BCL10*, *SPEN*, and *TP53* were significantly associated with a poor PFS (**Figure 6**). When analyzed in the gene set, mutation of genes involved in B-cell differentiation was associated with a poor OS and that involved in DNA damage response was associated with a poor PFS.

### Tumor Mutational Burden

The TMB in the pretreatment samples was not associated with the COO or tumor recurrence. The median TMB (range) values according to patient group were 12.2 (3.6–30.7) for ABC subtype, 20.7 (4.5–41.5) for GCB subtype, 12.6 (3.6–41.5) for cured, 14.4 (4.5–30.7) for refractory, 14.0 (5.4–39.7) for recurrent, and 10.8 (7.2–15.3) for late recurrent.

**Figure 7:**
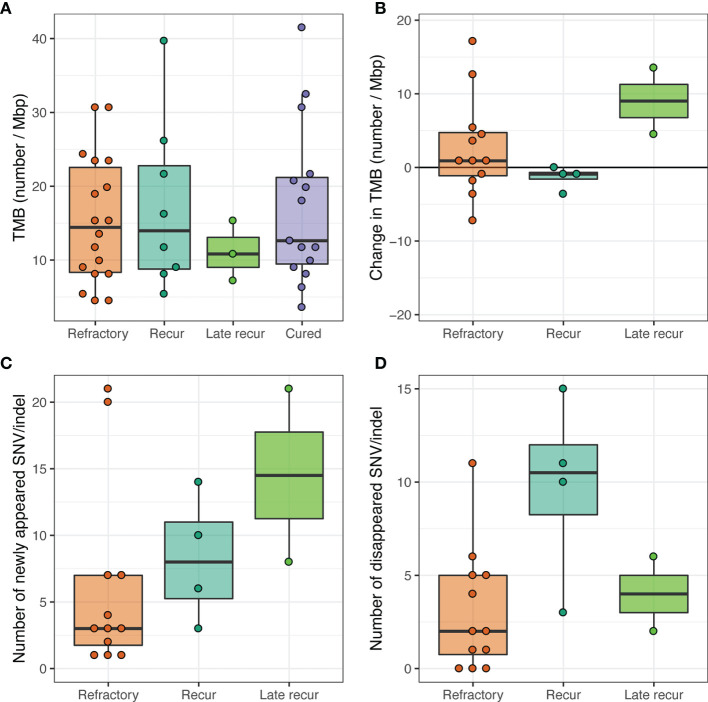
Tumor mutational burden (TMB). **(A)** TMB of pretreatment tumor samples. **(B)** Change in TMB between pretreatment and post-treatment paired tumor samples. **(C)** The number of SNV/indels that were not present in pretreatment tumor samples but appeared for the first time in post-treatment tumor samples. **(D)** The number of SNV/indels that were present in pretreatment tumor samples but disappeared in post-treatment tumor samples.

The survival rates were compared by dividing into two groups based on the median value of 13, with no difference in survival rates between low-TMB and high-TMB groups. The respective median PFS values (95% CI, in months) were 29.4 [14.7 to not reached (NR)] and 16.1 (8.1 to NR) (*P* = 0.400). The median OS values were 55.5 (22.3 to NR) and 29.6 (16.0 to NR). When changes in TMB were compared between pretreatment and post-treatment samples, patients with early or late relapsed DLBCL showed higher dynamics of subclones than those with refractory DLBCL, which may reflect the longer interval between biopsies. The TMB was low at the initial biopsy in late relapse patients, who showed high dynamics of subclones leading to increased TMB at the time of relapse (**Figure 7**).

### Clonal Evolution

**Figure 8:**
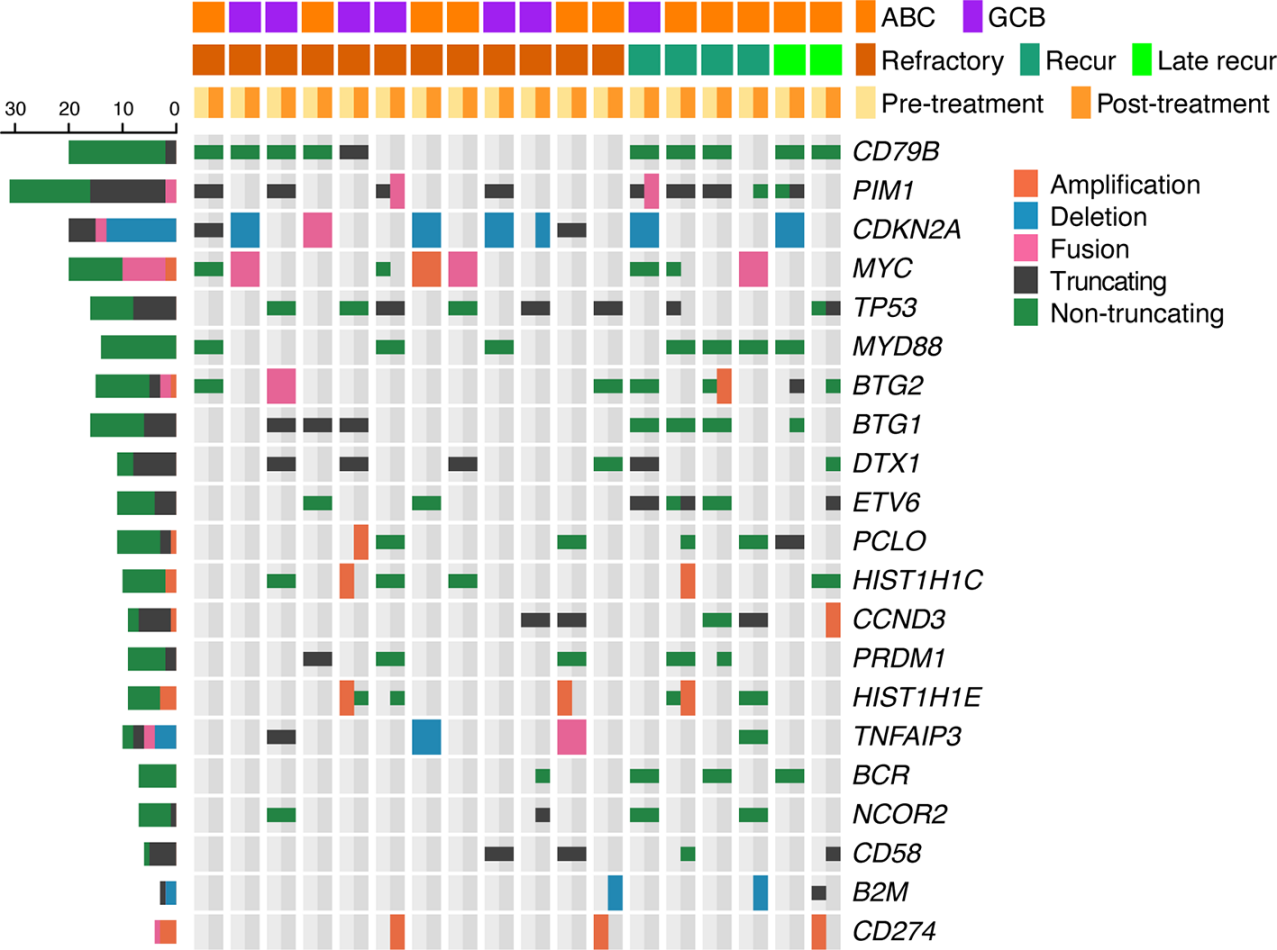
Mutational profiles of 18 paired pretreatment and post-treatment rrDLBCL samples. Genes mutated in > 3 cases are shown. The genes are ordered by the frequency of mutation. Unlike the **Figure 3**, copy numbers > 4 are marked as amplifications. COO, cell of origin; ABC, activated B-cell-like subtype; GCB, germinal center B-cell-like subtype.

**Figure 9:**
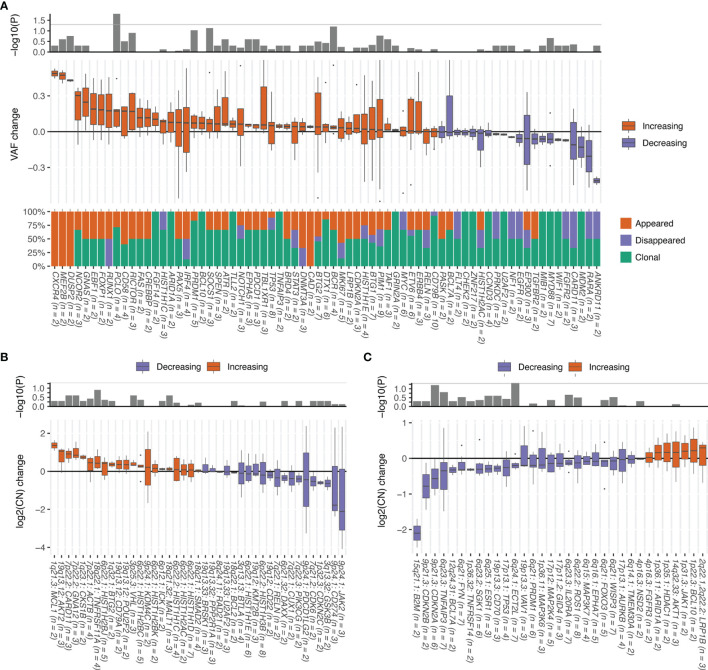
Change in alterations between pretreatment and post-treatment paired tumor samples. Genes altered in > 1 patient are included. *P* values were calculated using the Wilcoxon signed-rank test. Numbers of samples with alterations are shown in parentheses. **(A)** Change in variant allele fraction (VAF) of each gene and percentages of mutation SNV/indels that appeared for the first time in post-treatment tumor samples (newly appeared), disappeared in post-treatment tumor samples (disappeared), or were present in both pretreatment and post-treatment tumor samples (remaining). **(B)** Change in the log2 copy number of genes with amplification. **(C)** Change in the log2 copy number of genes with deletion.

To explore the mutational evolution, we compared 21 pairs of pre- and post-treatment samples from 18 patients. Median 11 (range, 4–33) SNV/indels are shared between pretreatment and post-treatment samples (**Figure 8**). To compare VAFs between samples with different purity, the VAF values were normalized to a median VAF value of 0.5. In some patients, *CXCR4*, *MEF2B*, and *DUSP2* mutations tended to appear for the first time in a major clone after chemotherapy. The VAFs of *NCOR2*, *GNAS*, *EBF1*, *FOXP1*, *RUNX1*, *PCLO*, *CD58*, *RICTOR*, and *CREBBP* mutations tended to increase after chemotherapy (**Figure 9A**, **Supplementary Table 8**). NF-κB-activating driver mutations such as *MYD88* and *CD79B* mutations were mostly truncal and changed little after the chemotherapy. Whereas Nijland et al. reported increased VAFs in *SOCS1* and *PIM1* (34) at relapse, only the VAF for *SOCS1* showed a tendency to increase in our data.

The copy numbers of amplified genes and deleted genes were also compared (**Supplementary Tables 9** and **10**). The copy numbers for *MCL1* (1q21.3), *AKT2* (19q13.12), *CARD11* (7p22.2), *GNA12* (7p22.2), *CKS1B* (1q23.1), *ACTB* (7p22.1), *TNFRSF11A* (18q22.1), *HIST1H2BJ* (6p22.1), *BTG2* (1q32.1), *CD79A* (19q13.12), *POU2F2* (19q13.12), *VHL* (3p25.3), and *HIST1H2BC* (6p22.1) tended to increase (**Figure 9B**). By contrast, the copy numbers of *B2M* (15q21.1), *CDKN2B* (9p21.3), *CDKN2A* (9p21.3), *TNFAIP3* (6q23.3), *BCL7A* (12q24.31), *FYN* (6q21), *TNFRSF14* (1p36.32), *SGK1* (6q23.2), *ESR1* (6q25.1), *CD70* (19p13.3), *TP53* (17p13.1), and *ECT2L* (6q24.1) tended to decrease (**Figure 9C**).

*CD58* mutation or *B2M* deletion, both of which are involved in the immune evasion, occurred for the first time in post-treatment samples in four of 18 (22%) patients. By contrast, amplification *of CD274* (PD-L1), which is also involved in the immune evasion, occurred for the first time in one patient but was lost in two patients. The one patient who lost *CD274* amplification (copy number = 9.7) gained *CD58* mutation. The other patient who lost *CD274* amplification (copy number = 5.1) gained *B2M* deletion. The patient who gained *CD274* amplification (copy number = 11.8) did not have *CD58* mutation or *B2M* deletion. Overall, seven (39%) patients gained or retained alterations involved in immune evasion after chemotherapy. Alterations in *TP53* and *CDKN2A* have been associated with poor survival outcome in previous studies (26, 35). Consistent with these results, the VAFs for the mutation of these genes tended to increase and the copy number of these genes tended to decrease.

### Molecular Classification

**Figure 10:**
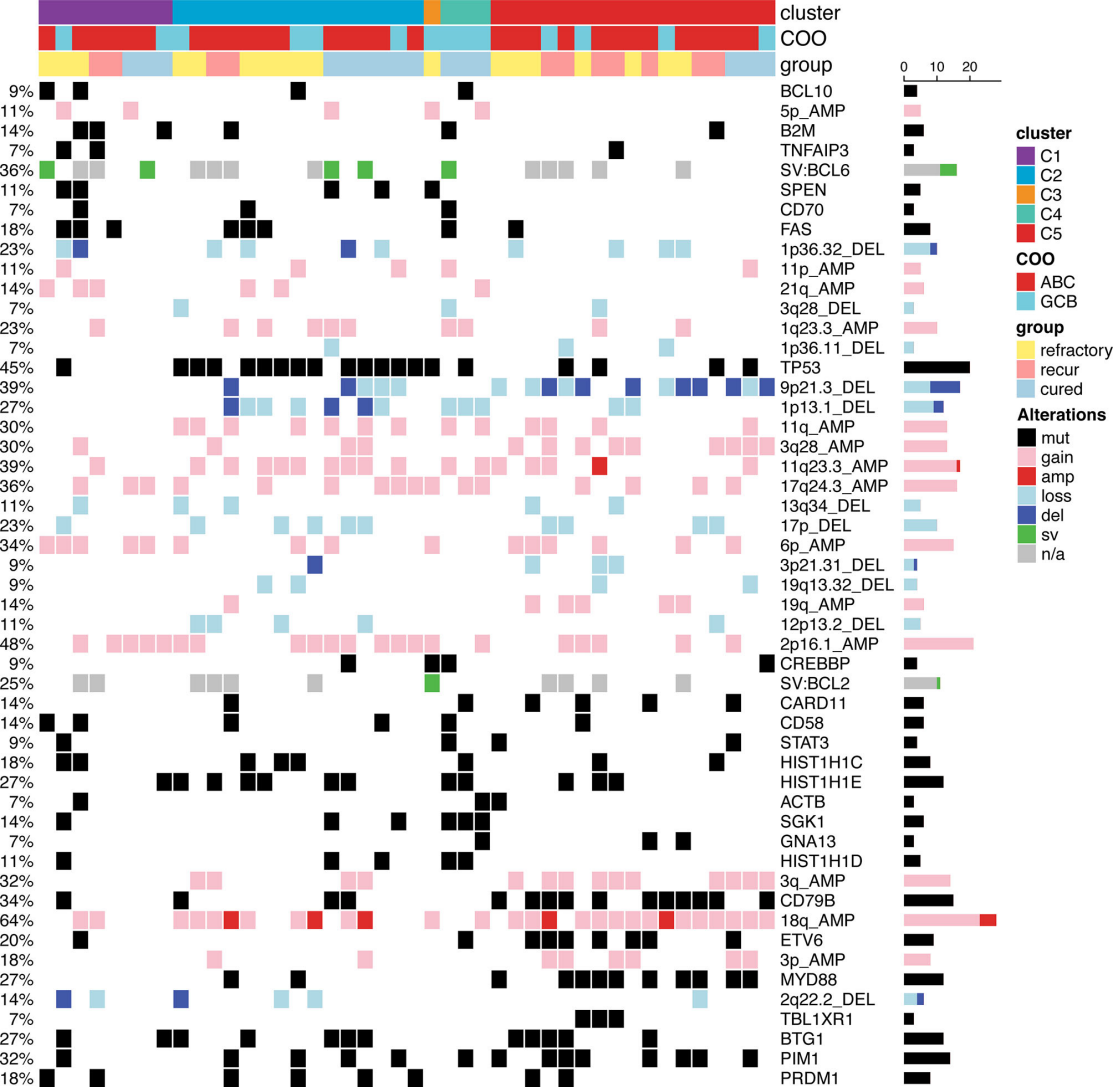
Molecular classification of pretreatment tumor samples according to the classification of Chapuy et al. (9). Among the 71 features used for classifications 51 features with > 2 alterations are shown. Features were ordered first by the cluster in which the feature contributed the most and second by the degree of contribution.

**Figure 11:**
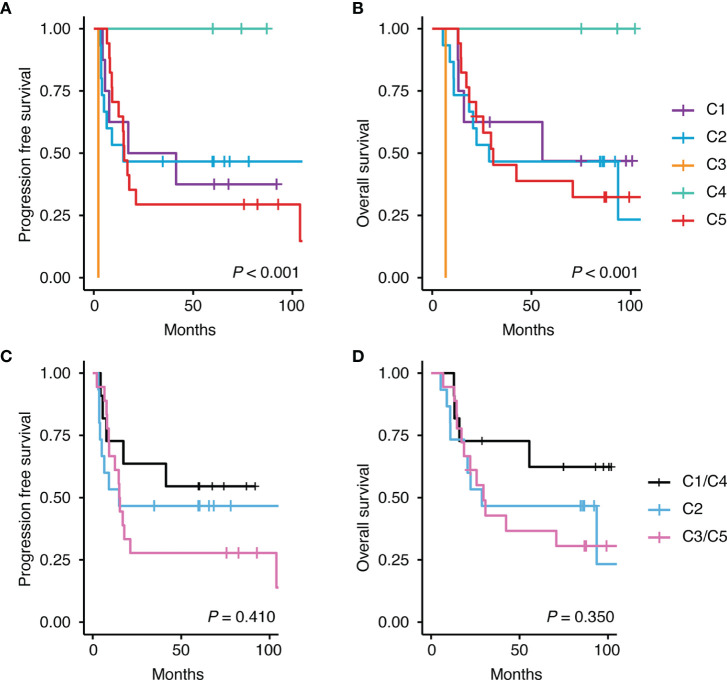
Differences in survival according to the classification of Chapuy et al. (9). Kaplan-Meier curves shows the difference in survival rate according to each class for **(A)** progression free survival and **(B)** overall survival. C1 and C4 were reported to have good prognosis and C3 and C5 were reported to have poor prognosis. This trend was also seen in our data on **(C)** progression free survival and **(D)** overall survival.

**Figure 12:**
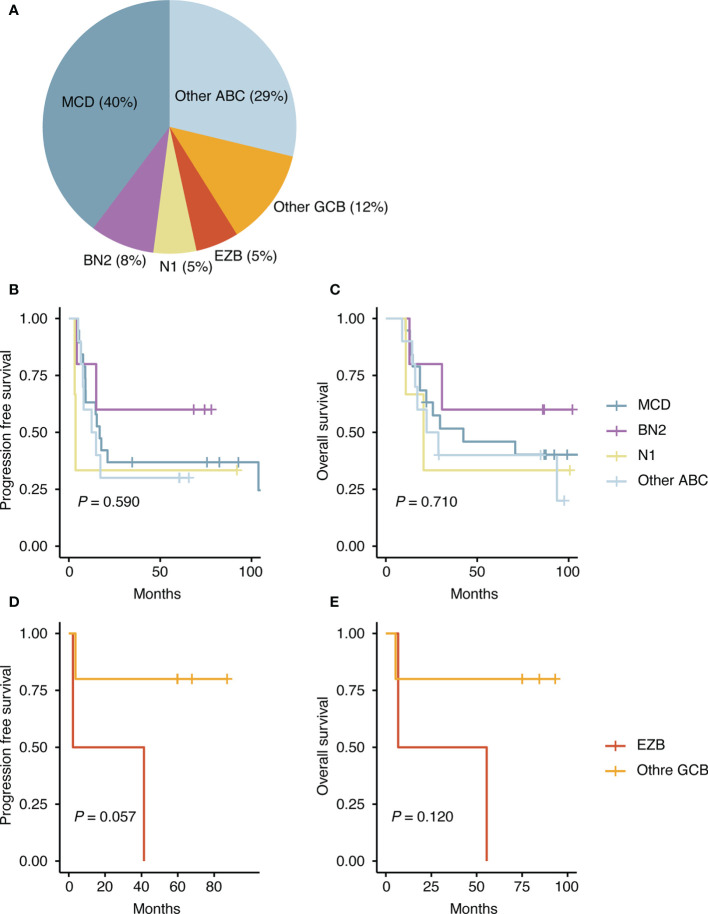
Molecular classification according to the classification of Schmitz et al. (8). **(A)** Proportion of assigned classes. **(B)** Difference in progression free survival among the ABC-type DLBCLs. **(C)** Difference in overall survival among the ABC-type DLBCLs. **(D)** Difference in progression free survival among the GCB-type DLBCLs. **(E)** Difference in overall survival among the GCB-type DLBCLs.

Several attempts have been made to classify DLBCL using mutational profiles (8, 9, 35), which have shown significant differences in survival rate between some subtypes. Because this molecular classification is made based on whole exome sequencing data, to use this molecular classification in clinical practice, it requires modification of the model, so that the tumors can be also classified using targeted panel sequencing data. We adjusted two published models (8, 9) and classified our DLBCL samples. When we applied the classification of Chapuy et al. to our data, only one patient was classified as C3, and C5 (**Figure 10**) accounted for a large percentage because of the higher frequency of the ABC subtype in our sample. Patients with C3 or C5 DLBCL had the worst survival (**Figure 11**), which was consistent with the data of Chapuy et al. (9). Similarly, when we applied the classification of Schmitz et al. to our data, the MCD subtype (40%) and other ABC subtype (29%) accounted for the main subtype. Among patients with DLBCL, the MCD subtype has the worst prognosis and other ABC subtype has an intermediate prognosis (8). Although statistical significance was not reached, we could reproduce the trend for survival difference in this molecular classification (**Figure 12**) which was classified using targeted panel sequencing data.

## Discussion

The genomic characteristics of tumors provide important information for selecting patients with poor prognosis for initial treatment and for developing further treatment plans. In this study, we examined the genomic profiles of post-treatment biopsy for 47 patients with rrDLBCL and compared the genomic profiles of pre- and post-treatment paired biopsies for 18 patients to identify gene candidates that may cause treatment resistance. In addition, using the pretreatment biopsy samples, we compared the survival rates between 29 patients with rrDLBCL and 15 cured patients in an attempt to identify genes with prognostic significance. Although the number of patients was not sufficient to overcome the genetic heterogeneity of DLBCL, we identified potential targets for treatment and made several compelling observations about tumor evolution.

The genome of rrDLBCL was enriched by mutation of genes involved in the cell cycle, NF-κB pathway, chromatin remodeling, RAS signaling, transcription regulation, DNA damage, B-cell differentiation, apoptosis, the PI3K–Akt–mTOR pathway, and immune evasion. As found in a previous study (24), the mutation frequency was higher for rrDLBCL than that reported for *de novo* DLBCL. Driver mutations such as those in *CD79B*, *CDKN2A*, *MYD88*, *MYC*, and *CCND3*, which are known to negatively affect the prognosis of DLBCL (36–40), were enriched in rrDLBCL. *CDKN2A* encodes two proteins, p16INK4a and p14ARF. P14ARF is a central actor in the cell cycle regulation process and participates in the ARF–MDM2–p53 and Rb–E2F1 pathways (41). Mutation in *CDKN2A* is the most common mutation associated with rrDLBCL and is an indicator of poor prognosis after R-CHOP treatment (42). The combination of *CDKN2A* and *TP53* mutations confers a worse survival independent of the IPI (25, 42). In our study, patients with *TP53* alterations had a worse prognosis, which was independent of the COO and IPI. Mutation of *TP53* has been reported in 17–23% of *de novo* DLBCL (25, 26) and 32% of rrDLBCL (24). In our cohort, the frequency of *TP53* was very high in both rrDLBCL patients and in the cured patients for whom all *TP53* mutations were disruptive mutations. Because of the high frequency of *TP53* alterations in the cured group, we initially questioned the effect of *TP53* on the survival of patients with DLBCL. However, the decreasing copy number of *TP53* and increasing VAF for *TP53* SNV/indels in the post-treatment biopsy support the idea that *TP53* alterations contribute to tumor evolution in DLBCL. This frequency of *TP53* variants, which was much higher than in other studies, may reflect the high coverage of our targeted sequencing or may be related to bias caused by the small number of samples in the cured group. Mutation of *PIM1* is the second most common mutation after *CDKN2A* in rrDLBCL and its mutation rate was slightly higher in ABC-type DBCL than in GCB-type DLBCL, as shown in a previous study (43). *PIM1* is a multifunctional gene that activates SOCS1 and SOCS3 to act as negative regulators of the JAK–STAT pathway (44). In addition, Pim-1 interacts with Myc (45–47), Bad (48), Bcl-2 (49), p21^Cip1/WAF1^ (50), p27^Kip1^ (51), RelA/p65 (52), and the fusion gene *E2A–PBX1* (53) to promote cell cycle progression, prevent apoptosis, and induce carcinogenesis. A recent study revealed that point mutations within the kinase Pim-1 reduce sensitivity to ibrutinib in ABC-type DLBCL (54). In our cohort, *PIM1* mutation frequency increased in the post-treatment biopsy, but *PIM1* mutation frequency was also high in the cured group and the overall VAF did not change in the post-treatment biopsy. Therefore, the impact of *PIM1* in rrDLBCL is questionable in our study.

Information about the mutational frequency at the initial diagnosis and in the post-treatment biopsy combined with changes in the VAF provide an insight into the clonal evolution and identification of genes with chemotherapy resistance. According to changes in the VAF of SNVs/indels, the mutations in rrDLBCL can be divided into three groups. The first group comprised mutations that were truncal and were maintained in both the initial and post-treatment biopsies with high dynamics of subclones. The genes showed partial loss of the major clone or acquisition of new variants at relapse. This was the most common pattern and may correspond to the late divergent mode of clonal evolution by Jiang et al. (55), which shows continuous alterations of the tumor genome with additional mutations acquired to achieve relapse. NF-κB-activating driver mutations, such as in *MYD88* and *CD79B*, were stable and changed little after immunochemotherapy. In the second group, which included *RUNX* and *DNMT3A*, the major mutation had completely disappeared in the post-treatment biopsy with the acquisition of a new variant. The third group included *CXCR4*, *MEF2B*, *DUSP2*, and *CAD*, and had a newly appearing clone at the post-treatment biopsy that was not detected in the initial biopsy. This new clone may have derived from a small clone at the initial biopsy whose presence was below the level detected by targeted sequencing or may represent an early divergent clone.

Among the genes altered in rrDLBCL, those with an increase in VAF or with a change in copy number in the post-treatment biopsy can be considered genes involved in relapse or chemoresistance. After immunochemotherapy, the frequency of mutations in genes, including *B2M*, *CD58*, *CARD11*, *CDKN2A*, *CDKN2B*, *PIM1*, *SOC1*, and *CREBBP*, increased. The mutations in some genes showed an increase in VAF (*NCOR2*, *GNAS*, *EBF1*, *FOXP1*, *RUNX1*, *PCLO*, *CD58*, *RICTOR*, and *CREBBP*) or an increase in copy number (*MCL1*, *ATK2*, *CARD11*, *GNA12*, *CKS1B*, *ACTB*, *TNFRSF11A*) or a decrease in copy number (*B2M*, *CDKN2B*, *CDKN2A*, *TNFAIP3*, *BCL7A*, *FYN*, *TNFRSF14*, *SGK1*, *CD70*, *TP53*). *PCLO* is the only gene with a significant increase in VAF, but the mutation is considered a passenger mutation and is frequent due to the large size of the gene (56). *CDKN2A* and *CDKN2B* showed increased overall frequencies of mutation and copy number loss. *CREBBP* encodes histone acetyltransferase; its mutation is a hallmark of GCB-type DLBCL and confers significantly worse OS and PFS (57, 58). *CREBBP* showed both increased frequency of mutation and increased VAF. NF-κB-activating genes show an increased mutation frequency and copy number (*CARD11*) and decreased copy number (*TNFAIP3*). It is noteworthy that immune-evasion genes showed increased overall mutation frequency (*CD58*, *B2M*) as well as increased VAF (*CD58*) and decreased copy number (*B2M*, *CD70*).

Refractory DLBCL refers to a tumor with primary resistance to the initial treatment, and patients show either no CR or a short duration of CR with relapse within 6 months. As shown in our data, refractory DLBCL is characterized by a relatively high TMB and close mutational distance between the pre- and post-treatment biopsy, that is, low dynamics of subclones. Of note is that, among the refractory DLBCL patients, those who did not experience CR had more mutations in the immune-evasion gene and more *DTX1* mutations than the other patients with rrDLBCL. There are studies suggesting the mutation of genes involved in antigen presentation as a resistance mechanism (55, 59). These findings support that immune evasion is important to refractoriness in rrDLBCL. Drugs to reverse immune evasion are actively being developed. In particular, drugs that target immunomodulatory molecules are promising. Several phase 1 or 2 clinical trials using anti-PD-1 or anti-PD-L1 monoclonal antibodies on DLBCL patients are ongoing (60). Some patients responded to the anti-PD-1 therapy, but the rate was low (61). The TMB is associated with genome instability and immunogenicity, and in concert with PD-L1 expression, has been shown to be a useful biomarker for immune check-point inhibitor selection across some cancer types (62–64). Few studies have examined the clinical impact of the TMB in malignant lymphoma, and further research on the prognostic impact of the TMB in lymphoma is needed.

In summary, rrDLBCL is an unresolved issue for DLBCL treatment and has attracted much attention in an attempt to identify rational targets for therapeutic intervention. We conducted targeted sequencing of samples from patients with rrDLBCL to understand better the molecular mechanisms relating to treatment resistance of rrDLBCL and to find candidate genes for the development of new treatments. A limitation of our study is that the number of patients may not have been sufficient to overcome the heterogeneity of the tumors. Despite this limitation, identification of candidate genes through identifying the mutational profile and integrative analysis of mutational evolution provide useful information for understanding the resistance mechanisms of rrDLBCL and for selection of therapeutic targets of rrDLBCL.

## Data Availability Statement

The original contributions presented in the study are included in the article/**Supplementary Material**. Further inquiries can be directed to the corresponding authors.

## Ethics Statement

The studies involving human participants were reviewed and approved by Institutional review board of Samsung Medical Center (IRB 2013-12-076-005). The patients/participants provided their written informed consent to participate in this study.

## Author Contributions

BL, HL, WSK, and YHK designed the study. BL, HL, JC, SEY, SJK, and W-YP collected and analyzed the data. BL and HL drafted the manuscript. WSK and YHK supervised and edited the manuscript. All authors had full access to all the data in the study and take responsibility for the integrity of the data and the accuracy of the data analysis. All authors contributed to the article and approved the submitted version.

## Funding

This work was supported by Intramural Research Program (Samsung 20x20 project) of the Samsung Medical Center [GFO2200161].

## Conflict of Interest

The authors declare that the research was conducted in the absence of any commercial or financial relationships that could be construed as a potential conflict of interest.
